# Immunotherapeutic Strategies for Gastric Carcinoma: A Review of Preclinical and Clinical Recent Development

**DOI:** 10.1155/2017/5791262

**Published:** 2017-07-11

**Authors:** Mohamed Abozeid, Antonio Rosato, Roberta Sommaggio

**Affiliations:** ^1^Department of Surgery, Oncology and Gastroenterology, Oncology and Immunology Section, University of Padova, Padova, Italy; ^2^Istituto Oncologico Veneto (IOV)-IRCCS, Padova, Italy

## Abstract

Gastric carcinoma (GC) is the 2nd most common cause of cancer-related death. Despite advances in conventional treatment and surgical interventions, a high percentage of GC patients still have poor survival. Recently, immunotherapy has become a promising approach to treat GC. Here, we present preclinical and clinical studies encouraging the use of vaccination, adoptive T-cell therapy (ACT), and immune checkpoint inhibitors, such as programmed cell death protein 1 (PD-1)/programmed death-ligand 1 (PD-L1) or cytotoxic T-lymphocyte-associated protein 4 (CTLA-4). The ongoing immunotherapy clinical trials have shown promising results in safety and tolerability even in late-stage GC patients. Moreover, we highlight that the combination of ACT with chemotherapy could be the best choice to treat GC.

## 1. Introduction

GC is the fourth most common cancer in the world and the second most common cause of cancer-related death [1]. Radical surgery remains the first curative choice, while perioperative chemotherapy is a standard treatment in early GC [2, 3]. However, 50% of advanced GC patients suffer from local or systemic recurrence even after standard adjuvant treatment, and only 10–15% of all GC patients achieve 5-year overall survival (OS) [4, 5].

Today, immunotherapy has important clinical applications with potential favorable outcomes and limitations. Common obstacles are the generation of immune effectors, safety, and applicability to a large number of patients. In this regard, it is critical to understand how cancer cells behave and interact with surrounding components in the tumor microenvironment such as parenchymal cells and inflammatory cells including lymphocytes and extracellular matrix (ECM) [6, 7] and the role these elements have in tumor survival, proliferation, and metastasis [6]. In tumor microenvironment, cancer cells release cytokines that modify the microenvironment contexture, while noncancer cells secrete cytokines and growth factors that affect both tumor growth and behavior, such as invasion and metastasis [7]. In this dynamic microenvironment, cells interact, which leads to tumor progression.

GC microenvironment is infiltrated with tumor infiltrating lymphocytes (TILs), which have a more pronounced cytolytic activity than stromal T-cells in chronic gastritis, and the high levels of TILs could be considered a good prognostic factor [8].

The oncogenic bacteria* Helicobacter pylori* (*H. pylori*) promote gastric chronic inflammation that contributes to intestinal metaplasia development and oncogenic mutations in GC by downregulating immune reactions through interference with antigen presentation, inactivation of T-cell proliferation, and fostering of T-cell apoptosis partially via human interaction domain 2 (VacA) [8, 9]. Accordingly, in vivo studies have proposed that type 1 T helper cells (Th1) have a main role in controlling* H. pylori* through cytokine release, B-cell activation, and production of antibodies [9]. Therefore, in the absence of Th1 cytokines, such as interferon-gamma (IFN-*γ*), both gastric atrophic changes and prolonged inflammatory response are abrogated [9].

Here, we will review current research and application of immunotherapy in GC, also focusing on novel therapies with immune checkpoint inhibitors such as the monoclonal antibodies (mAbs) to PD-1/PDL1 or CTLA-4.

## 2. Immunotherapy in GC

Malignant cells can express many different proteins that are potentially recognizable by the immune system; nonetheless, tumors develop immune regulatory circuits with immunosuppressive effects on the cancer environment which interfere with the antitumor response [10]. Immunotherapy represents a therapeutic opportunity capable of modulating the host immune system to fight cancer with less toxicity than conventional chemotherapy [10]. Recently, immunotherapy has shown satisfactory clinical results in patients with advanced cancers treated with vaccination, ACT, and/or checkpoint inhibitor mAbs.

## 3. Vaccination in GC

The main role of cancer vaccines is to activate and expand tumor associated antigen- (TAA-) specific T-cells, thus enhancing the antitumor immune response through activation of preexisting immunity, initiation of unprecedented immunity, or strengthening of the current immune response. Several vaccination studies have been performed to enhance immune responses against GC. Dendritic cells (DCs) are antigen presenting cells (APCs) that can activate natural killer (NK) cells, B-cells, and naïve and memory T-cells [11, 12]. Despite having a promising role in cancer vaccination, the use of DCs is limited in clinical trials due to their short life span. Some studies in GC patients have demonstrated the correlation between DC numbers and clinicopathological status and prognosis, where patients with more DC infiltration had less lymph node (LN) involvement and better OS [13–15]. A study where DCs from advanced gastrointestinal tumor patients were pulsed ex vivo with melanoma-associated antigen (MAGE) A3 peptides (expressed also in GC-56-REF) showed an improvement in performance status in 4 patients, while 3 additional patients had minor tumor regression without direct correlation between outcome and immune response [16]. In a phase I clinical trial, 9 advanced or recurrent GC patients with tumors overexpressing the human epidermal growth factor receptor-2 (HER2)/neu received a regimen of DCs pulsed with HER2(_p369_) peptide. Vaccine was well tolerated and induced tumor specific T-cell response, with partial clinical response and decrease in carcinoembryonic antigen (CEA) marker in one patient and stable disease for 3 months in another patient [17]. Regimens of cancer vaccines associated with chemotherapy showed promising results in GC patients. In radically resected stage III/IV GC, a combination of adjuvant Bacille Calmette-Guérin (BCG) vaccine with chemotherapy resulted in a prolonged 10-year OS (47.1%) as compared to monochemotherapy (30%) or surgery alone (15.2%) [18]. In a phase II clinical trial involving patients with advanced GC and gastroesophageal junction (GEJ) adenocarcinoma, the gastrin-17 diphtheria toxoid (G17DT) vaccine targeting gastrin peptide in association with cisplatin and fluorouracil (5-FU) chemotherapy led to a longer time-to-progression (TTP in 69% of patients considered immune responders and a better OS compared to the nonimmune responder patients) [19]. Recently, a phase I clinical trial by Higashihara et al. demonstrated the safety of HLA-A*∗*2402-restricted URLC10-A24-177 and vascular epidermal growth factor receptor (VEGFR1-A12-9 1084) epitope peptide cancer vaccines in 14 chemotherapy-resistant advanced GC patients. Specific cytotoxic T-lymphocytes (CTLs) positive responses were determined in 62.5% and 50% of patients for URLC10 and VEGFR1, respectively [20].

## 4. Preclinical Studies of ACT in GC 

GC has different precursor events such as* H. pylori*, atrophic gastritis, and intestinal metaplasia and dysplasia [21] with a multistep carcinogenesis including genetic variants and molecular abnormalities that lead to a malignant transformation of the gastric mucosa [22–24]. The cofactors involved in GC pathogenesis are still unknown and the detailed mechanism of cancer development is uncertain [25].

GC adenocarcinomas are histologically classified according to the 2010 WHO classification [24] into four major subtypes: tubular, mucinous, papillary, and poorly cohesive and uncommon variants.

Each GC subtype has its featured genetic profile and molecular diversities. Targeting the specific molecular abnormalities could prevent tumor cells from skipping the host immune system and also predict the prognosis. Hence, genetic and molecular studies are needed to understand different pathognomic molecular expressions in GC cells and distinguish which subtype will benefit from immunotherapy [22, 26].

NK cells have cytotoxic activity against solid tumors including both allogeneic and autologous derived GC cells lines [27] and could prevent cancer metastatic dissemination [28]. A high NK cell level, demonstrated by the expression of CD57 antibody in 146 GC tissue sample, was associated with smaller tumors, less LN involvement, a higher rate of surgical care, and a better 5-year OS [29], indicating a possible prognostic role of these cells in GC. Nie et al. used different HLA-A matched allogeneic GC cells to stimulate peripheral blood lymphocytes from GC patients or from healthy donors and assessed them against different cell lines. Induced CTLs had antitumor effects against HLA-A2 and HLA-A24 GC cell lines with no effect against HLA-A2 negative GC cells or any other cancer cells [30]. When TILs and specific T-cells from peripheral blood of GC patients are expanded in vitro, they show specific type 1 T-cells response to GC antigens. This would reduce tumor growth; however, Th1/Tc1 response would be enhanced by vaccination with the appropriate cancer peptides or by injection of the autologous tumor peptide-specific T-cells expanded in vitro [31].

In addition, Kono et al. isolated major histocompatibility complex-1 (MHC-1) restricted T-cells specifically binding to GC antigens from primary tumors, metastatic LNs, and ascites of autologous GC, which showed different recognition patterns towards GC antigens [32]. Fujie et al. succeeded in using splenic MAGE-specific CTLs targeting HLA-A2 cancer cells, an antigen expressed in testis and several cancers including GC, pointing out the role of spleen in ACT in GC [33]. Cytokine induced killer cells (CIK), as well as other interesting immune competent cells, are considered a good choice in ACT in different tumors [34–37]. CIK cells are a heterogeneous population of immune effector cells generated after culturing lymphocytes with an anti-CD3 antibody and other cytokines such as IFN-*γ* and interleukin-2 (IL-2) in vitro with a high proliferative activity and antitumor cytotoxic effect [38]. CIK cells have antiproliferative and antiapoptotic activity against the MGC-803 GC cell line [39] and the MKN74 human GC cell line, mainly releasing IFN-*γ* and tumor necrosis factor-alpha (TNF-*α*). MKN74 tumor bearing nude mice injected with 3 million and 10 million CIK cells showed 58% and 78% tumor reduction, respectively [40].

ACT is recommended in combination with chemotherapy due to difficulty in GC stroma infiltration as shown in in vivo studies [41, 42].

Besides its cytotoxic effect through inhibition of DNA synthesis and transcription, oxaliplatin can also induce an immunogenic cancer cell death (ICD) triggering the high-mobility group box 1 protein to induce T-cells against tumor cells [43]. Therefore, the combination of CIK cells with oxaliplatin against drug resistant GC in in vitro and in vivo experiments resulted in a release of large amounts of cytokines, such as IL-2, with a significant antitumor effect compared to monotherapy with chemotherapy or CIK cells only [44].

T-cell depleting chemotherapy would improve ACT efficacy as host immunosuppression status prolongs the persistence of endogenous T-cells in circulation, while reducing autoimmune reactions on normal tissue. However, patients have severe toxicities due to infectious complications [45]. Thus, Kobold et al. improved ACT efficacy in a GC mouse model without depleting T-cells by addressing T-cell recruitment to tumors. Simian virus 40 (SV40) T antigen-specific T-cells were transduced with a truncated human epidermal growth factor receptor (EGFR) as a marker protein. The combination of ACT with an anti-EGFR, antiepithelial cell adhesion molecule (EpCAM) bispecific antibody (BiAb) that selectively recognizes transduced T-cells increased T-cell infiltration of tumors, reduced tumor growth, and prolonged survival when compared to ACT only or control antibody [46].

Du et al. studied the biodistribution and antitumor effects of CIK cells via peritumoral, intravenous (I.V.), and intraperitoneal routes in GC mice model. Only a limited number of CIK cells succeeded in reaching the tumor via I.V. and intraperitoneal routes, while peritumoral injection showed high accumulation of CIK cells in the tumor site for 48 hours with a better antitumor response. This indicates that peritumoral injection of effector cells represents an effective delivery method of ACT with a minimally invasive surgical procedure [47].

## 5. Clinical Studies of ACT in GC 

Activated T-lymphocytes showed promising results against several malignancies in several clinical trials [48]. Some clinical trials evaluated the efficacy of ACT when combined with chemotherapy in advanced GC patients.

Zhang et al. evaluated the prognostic role of expanded activated autologous lymphocytes (EAALS) stimulated by anti-CD3 mAb (OKT3) and IL-2 in GC patients. 42 GC patients who received EAALS had a better OS than the control group that received conventional treatment only (*p* = 0.028) [49]. In a randomized clinical trial, T-activated lymphocytes (TALs), extracted from patients, expanded in vitro with IL-2, and stimulated with autologous tumor, were administered either intraperitoneally or intravenously to 44 advanced GC patients in combination with chemotherapy (low-dose cisplatin and 5-FU) to evaluate the survival benefit. Patients receiving the combined treatment showed a marked improvement in OS compared to those who received chemotherapy only (*p* < 0.05) [50].

Jiang et al. evaluated the combined regimen of CIK cells with chemotherapy (FOLFOX4) in 32 advanced GC patients after palliative gastrectomy. In comparison with the control group (FOLFOX4 only), the combined regimen had a marked reduction of tumor markers, higher total remission rate (56.3% against 48%), and better quality of life (QoL) but no differences in 2-year OS [51]. To evaluate the possible toxicities of combining ACT and chemotherapy in GC elderly patients, Jäkel et al. assessed a regimen of chemotherapy (FOLFOX) followed by autologous CIK cells. Side effects were not severe and were reversible, and patients had a better total remission rate [52]. These results motivate more studies on combining CIK cells with chemotherapy in advanced GC to confirm the effects on OS.

In a clinical trial, GC patients received a combination of autologous NK cells, *γ*  *δ* T-cells, and CIK cells with chemotherapy. Two-year progression free survival (PFS) improved significantly and the regimen was well tolerated with better QoL but with no statistically significant difference in 2-year OS [53]. Wada et al. performed a pilot study, where 7 patients received gamma delta T-cell type (V*γ*9V*δ*2) with zoledronate intraperitoneally as a local treatment for malignant ascites in advanced GC; a marked reduction in the number of peritoneal malignant cells and ascetic volume was observed with no marked or irreversible side effects [54]. In another trial, a regimen of capecitabine and oxaliplatin in combination with CIK cells administered intraperitoneally in GC malignant ascites showed a marked improvement of malignant ascetic volume and OS with low side effects [52].

Other clinical trials were performed to evaluate the ACT/chemotherapy combination in R0 postsurgically resected GC patients. A combination of CIK cells and chemotherapy was used in stage II/III GC after radical gastrectomy. A marked benefit was noticed with significant difference in 5-year OS compared to the control group that received chemotherapy alone (56.6% versus 26.8%, *p* = 0.014) and no marked side effects were noted [55]. Shi et al. conducted a clinical trial evaluating autologous CIK cells with chemotherapy (5-FU backbone) in 151 stage III/IV (M0) GC patients after (R0/D2) gastrectomy. Results showed a significant improvement in both 5-year OS (32.4%, *p* = 0.071) and 5-year disease-free survival (DFS) (28.3%, *p* = 0.044) compared to the monochemotherapy control group [56].

A clinical trial evaluated the possible toxicities of ACT/chemotherapy regimens in GC patients. After R0/D2 gastrectomy, 89 stage II/III GC patients received autologous CIK cells plus 5-FU or capecitabine backbone chemotherapy. Only 23.6% of patients had grade I/II side effects such as fever, fatigue, rash, and diarrhea, while none suffered from grade III/IV side effects or an autoimmune response. In addition, the regimen showed improvement in DFS (*p* = 0.006) and OS (*p* = 0.028) [57].

## 6. Ongoing Clinical Trials of ACT in GC

Currently, several ongoing clinical trials use ACT in different advanced solid tumors including GC. A regimen of preconditioning chemotherapy (cyclophosphamide/fludarabine) and anti-PD-1 mAb is administered followed by I.V. infusion of in vitro expanded autologous TILs and IL-2 [58]. In a current clinical trial, chimeric antigen receptor (CAR) T-cells specific for EpCAM were infused into relapsed/refractory GC patients evaluating CAR T-cell safety and efficacy [59].

Currently, a phase I/II clinical trial is investigating the cytotoxic activity of engineered pluripotent stem cells (iPIK) and T-cells, which specifically bind to HER2 of GC in patients with liver metastasis [60]. In a current clinical trial also targeting HER2 in GC, the safety and efficacy of therapy with trastuzumab and NK cells are being evaluated. Patients receive both trastuzumab and NK cells in the first cycle and then trastuzumab for another 3 cycles, except for patients with a tumor response after 2 cycles who then receive NK cells in the fourth cycle [61]. Another clinical phase I trial assesses the safety of bispecific antibody armed autologous T-cells (HER2Bi-Armed T-cells) in GC and esophageal cancers [62].

Currently, a phase I/II clinical trial assesses CAR T-cells specifically targeting mucin 1 (MUC1) in solid tumors including GC, as its overexpression interferes with chemotherapy leading to refractory cancers [63].

In a current phase I/II clinical trial, advanced metastatic GC and GEJ cancer patients receive a combination of S-1 (5-FU prodrugs tegafur, gimeracil, and oteracil) and dendritic cell activated CIK (DC-CIK) [64].

A current phase I/II clinical trial is assessing adoptive *γ*  *δ* T-cell and CIK cell therapy by monitoring drug related toxicity in stages II-IV GC patients [65]. In a current phase 1b clinical trial, anti-CEA CAR T-cells are injected into the hepatic artery targeting hepatic metastasis from GC expressing CEA as TAA [66].

Other clinical trials are evaluating regimens of ACT and chemotherapy after oncosurgical intervention in advanced GC patients [67]. In one such phase II trial, a regimen of autologous tumor lysate-pulsed dendritic and CIK cells (Ag-D-CIK) and chemotherapy is currently being evaluated in stages I-III GC after radical gastrectomy [68].

## 7. Preclinical Studies of Checkpoint Inhibitors

CTLA-4 and PD-1 are T-cell inhibitory receptors known as checkpoint molecules that play a critical role in immune inhibition. Due to its higher affinity, CTLA-4 competes with CD28 on T-cells for receptors CD80 and CD86 on APCs interfering with T-cell activation downregulating the immune response [69–71]. PD-1 is expressed on activated T-cells, NK cells, and B-cells, while the transmembrane protein PD-L1 is expressed on several immune cells and tumor cells in the presence of inflammatory mediators. PD-1/PD-L1 axis is dynamically active in peripheral tissue to control inflammatory reactions [72], while, in malignancy, PD-1 on activated T-cells binds to PD-L1 on tumors providing tumor escape and subsequent tumor progression [73, 74]. PD-1/PD-L1 overexpression has been observed in numerous malignancies including GC, and restoration of antitumor T-cell activity by targeting checkpoint molecules has been demonstrated in several studies [75]. Currently, different studies are trying to better understand the genetic and molecular pathways of checkpoint molecules to develop targeted mAbs in GC, which is considered a good candidate for this field of study [76, 77].

## 8. Genetic Studies of Checkpoint Inhibitors 

Aberrant PD-1 expression was determined in GC, provoking its role in tumor skipping from the immune system. Several studies have demonstrated a possible connection between PD-1 or CTLA-4 polymorphism and GC development [78–82].

Savabkar et al. analyzed DNA of 122 GC using polymerase chain reaction-restriction fragment length polymorphism (PCR-RFLP) assay, showing high frequencies of PD-1.5CT genotypes in GC (*p* = 0.026) [78]. Tang et al. extracted DNA from lymphocytes and used ligation detection reaction (LDR) to detect polymorphisms. The study, which involved analysis of three single nucleotide polymorphisms (SNPs) in newly diagnosed 330 gastric cardia adenocarcinoma (GCA) patients, revealed a possible correlation between GCA and PD-L1 SNPs (PD-1 rs2227982 C>T type) [79]. Hayakawa et al. reported a patient with an autosomal dominant immune dysregulation syndrome developed from CTLA-4 haploinsufficiency. When the patient was 34 years old, he developed multifocal poorly differentiated GC with atrophic gastritis, the same condition observed in at least 2 other patients, suggesting a role of autosomal dominant immune dysregulation syndrome due to CTLA-4 haploinsufficiency in GC development [83]. In 2014, Kordi-Tamandani and his group pointed out the role of CTLA-4 gene promoter hypermethylation as a risk factor in developing GC. CTLA-4 gene methylation was markedly correlated with GC when compared to the unmethylated gene (OR = 4.829; 95% CI: 2.46–9.48; *p* < 0.001) and the CTLA-4 expression profile was markedly higher in GC tissue samples than in normal tissue on the tumor margins [84].

## 9. PD-1/PD-L1 and CTLA-4 Expression and Prognostic Role 

Several studies revealed high PD-L1 expression on GC, suggesting a possible response to a PD-L1 mAb therapy. PD-L1 is 50% expressed in Epstein-Barr virus (EBV)^+^ GC tumor cells and 94% in immune cells, while in EBV^−^ GC the PD-L1 expression was positive only when associated with microsatellite instability (MSI), suggesting that patients with EBV^+^ and MSI GC may have better response to PD-1 blocking therapy [85]. Furthermore, Saito et al. confirmed that PD-1 expression on CD8^+^ and CD4^+^ T-cells in GC is higher compared to normal gastric mucosa [86].

CD8^+^ T-cells, isolated from GC tissue samples and peripheral blood mononuclear cells (PBMCs), markedly expressed PD-1 in GC patients compared to healthy donors. Studies that evaluated PD-1/PD-L1 role as a prognostic factor and its correlation with clinicopathological status showed controversial results. Although some studies revealed PD-L1 expression as a predictive marker for a PD-L1 mAb therapy, other studies revealed a tumor response to PD-L1 therapies with no PD-L1 expressing malignant cells [87, 88]. Sun et al. detected PD-L1 expression in 42.2% of GC tissues with no expression in normal gastric and gastric adenoma samples. PD-L1 expressing GC was associated with an increase in tumor size (*p* < 0.05), LN involvement (*p* < 0.01), and deep invasion (*p* < 0.01). PD-L1 was expressed in fresh isolated T-cells while it was less expressed in B-cells and DCs [89] and one of these mAbs dampened PD-L1 inducing T-cell apoptosis [89]. Schlößer et al. evaluated PD-1 and PD-L1 expression in GC tumor microenvironment and regional LNs [90]. Nearly half of GC patients (44.9%) expressed PD-L1 in tumor microenvironment which contained high numbers of TILs. PD-L1^+^ primary tumors were associated with 100% regional LN involvement. Additionally, mean OS in PD-L1^+^ was markedly lower than in PD-L1^−^ patients (39.1 months versus 54.2 months, *p* = 0.011), indicating the role of PD-L1 as an independent worse prognostic factor in GC (*p* = 0.024) [90]. In 34 newly surgically resected GC and GEJ adenocarcinoma samples, PD-L1 was expressed in 12% of malignant cells and in 44% of tumor microenvironment nonmalignant cells. Samples dense with CD8^+^ T-cells showed higher PD-L1 expression in both malignant and nonmalignant stromal cells with a decrease in PFS and OS [91]. No correlation was found between PD-L1 expression and staging, indicating that inhibition may occur in early stages as well as late stages of disease [91]. The study by Chang et al. revealed a marked correlation between PD-1/PD-L1 expression in tumor cells and TILs of GC and clinical progression, namely, advanced tumors (*p* < 0.001), LN involvement (*p* < 0.001), and perineural invasion (*p* < 0.001). In TILs, CD8^+^ T-cells with high PD-L1 expression had a lower 5-year OS (*p* < 0.001); thus, their expression as an independent prognostic factor in 5-year OS is still controversial [92].

Another study considered PD-L1^+^ T-cell increase as a poor prognostic factor in GC. Immunohistochemistry (IHC) analysis performed in 132 stage II/III GC after surgical resection showed PD-L1^+^ expression in 50.8% of samples, especially in tumors larger than 5 cm (*p* = 0.036) with low 5-year OS (*p* < 0.001) [93]. An IHC study correlated PD-L1 expression to a poor 3-year DFS (*p* < 0.05), enlarged tumors (*p* = 0.046), and lymphatic invasion (*p* = 0.007) [94].

In addition, PD-L1 expression was correlated with tumor invasion (*p* = 0.004) and poor survival (*p* = 0.017) in GC patients. In this study, tumor invasion was determined using the contrast enhanced ultrasonography (CEUS). CEUS has several advantages; it is a well-tolerated noninvasive technique in contrast to the standard invasive upper gastrointestinal endoscopy and has a smaller ionizing burden than a computed tomography (CT) scan. This study pointed out the promising role of this imaging technique in predicting PD-L1 expression (*p* = 0.0003) [95]. A recent meta-analysis comprised 10 studies with 1901 GC patients assessing PD-L1 expression, low OS (*p* = 0.01), and poor clinicopathological status [96]. In contrast to previous studies, more recent studies showed that PD-L1 expression in GC may be a good prognostic factor. Böger et al. studied PD-1 and PD-L1 expression in 465 GC and 15 hepatic metastasis tissue samples. Results correlated with the high PD-L1 expression in tumor and immune cells and the better OS [73]. In another study, the high circulating PD-L1 expression in 80 advanced GC patients showed a marked correlation with LN involvement (*p* = 0.041) and a statistically significant better 5-year OS (*p* = 0.028) [97]. In addition, Kim et al. involved 243 GC patients who underwent radical oncosurgical resection, revealing a favorable role of PD-L1 expression as a prognostic factor [98]. In the above-mentioned study by Schlößer et al., CTLA-4 expression was also evaluated in tumor microenvironment and regional LNs in 127 GC patients. Positive CTLA-4 expression was revealed in the tumor microenvironment in 86% of patients; it had low expression in TILs but a strong correlation between its positive expression and poor OS (*p* = 0.018) and between its negative expression and the high grading and diffuse type malignant cell occupation (*p* = 0.012 and *p* = 0.006, resp.). Also, CTLA-4^+^ primary tumors are associated, in most cases, with positive LN involvement. Yet, the CTLA-4 expression is not considered as an independent prognostic factor (*p* = 0.062) [90].

## 10. Clinical Trials of Checkpoint Inhibitors

Up to now, most GC clinical trials involving checkpoint inhibitors are phase I and II trials. Takaya et al. evaluated PD-1^+^ T-cells levels before and after gastric resection in 33 GC patients, showing higher PD-1^+^ T-cell expression after surgical resection [77]. Therefore, according to this study, the use of checkpoint inhibitors as adjuvant chemotherapy after gastric resection is recommended in more trials as the surgical stress could upregulate PD-1^+^ T-cell levels inhibiting the immune response. A multicenter study evaluated anti-PD-L1 adverse effects in a phase I clinical trial when applied to patients with different solid tumors, including 7 GC patients. The majority of patients (61%) suffered from side effects, mostly low grade, such as fatigue, nausea, diarrhea, and headache, while only 9% of patients suffered from grade III/IV side effects. However, 39% of patients had related immune toxicity, including hypothyroidism and hepatitis [99]. A phase II clinical trial by Ralph et al. showed a low objective response rate when anti-CTLA-4 mAb tremelimumab was administered in 18 locally advanced/metastatic GC and esophageal cancer patients as a second-line treatment after failure of cisplatin backbone chemotherapy. Patients received varying numbers of tremelimumab cycles every 3 months. Drug was tolerable with mild toxicities and only a single death due to intestinal perforation resulting from autoimmune colitis. Antitumor response was evaluated in four patients who had stable disease and one patient who achieved partial response in the period between 25.4 months and 32.7 months after the beginning of treatment [100]. In a case study, a 64-year-old stage IIA GC patient underwent subtotal gastrectomy, had a recurrence, and subsequently received conventional chemotherapy with trastuzumab and pertuzumab. He had no clinical response. With pembrolizumab every 3 weeks, he achieved partial response with no drug related toxicity and a marked decrease in CEA levels. In this patient, IHC and PCR studies showed PD-L1^+^ and proficient mismatch repair (pMMR)^+^. This is the first study showing pMMR/microsatellite stability response to anti-PD-L1 mAbs in GC patients [101].

## 11. Ongoing Clinical Trials of Checkpoint Inhibitors 

Recently, ongoing phase I/II clinical trials use the combination of checkpoint inhibitors nivolumab and ipilimumab or monotherapy with nivolumab in advanced GC and GEJ cancer patients; MEDI4734 and tremelimumab are being used in another trial [102, 103]. Up to date, results of the first trial showed nivolumab to be a well-tolerated drug with antitumor efficacy in advanced GC and GEJ adenocarcinoma [104]. Another ongoing phase III study compares the combination of nivolumab and ipilimumab with the combination of nivolumab and chemotherapy in advanced GC and GEJ adenocarcinoma patients [105]. In other studies, anti-PD-L1 mAbs are being evaluated as a monotherapy and compared with conventional chemotherapy in GC. Monotherapy nivolumab is currently being assessed in a phase III clinical trial in advanced GC and GEJ cancer patients and atezolizumab is currently being assessed in a phase I clinical trial [106, 107]. Currently, nivolumab is the first immunotherapy treatment for advanced GC and GEJ cancer patients in phase III trial, achieving marked improvement in OS (*p* < 0.0001) and PFS (*p* < 0.0001) [108].

Nivolumab is also being investigated as an adjuvant monotherapy in resectable GEJ cancer patients [109]. Anti-PD-L1 avelumab is currently being investigated in a phase I clinical trial against different advanced solid tumors including GC and GEJ cancer, and the preliminary results show a safe and tolerable drug in treated patients [110, 111]. An ongoing phase III clinical trial currently compares pembrolizumab (MK-3475) and paclitaxel as a second-line treatment in advanced GC and GEJ cancer after a first-line failure with platinum or 5-FU [112]. Another ongoing phase 1b trial is assessing the antitumor effect and safety of pembrolizumab in different solid tumors including PD-L1^+^ GC, and preliminary results reveal its controllable toxicity and effective cytotoxicity against advanced GC patients [113, 114]. Anti-PD-L1 (avelumab) is compared with conventional chemotherapy as a first- and third-line treatment in advanced GC and GEJ cancers in phase III trials [115, 116].

In a phase II clinical trial, ONO-4538 (nivolumab) combined with chemotherapy is assessed in advanced and recurrent GC [117]. In another phase I/II study, nivolumab was evaluated as monotherapy and in combination with chemotherapy against EBV^+^ GC [118]. In a phase I/II clinical trial, pembrolizumab is involved in a neoadjuvant treatment plan, which includes chemotherapy and radiotherapy in resectable GCA and GEJ (cancer stages IB-IIIB) [119]. Pembrolizumab combined with trastuzumab and chemotherapy in HER2^+^ GC patients is being evaluated in another phase I/II clinical trial [120]. Pembrolizumab (MK-3475)/chemotherapy or monotherapy pembrolizumab is currently being assessed in clinical trials phases II and III in advanced GC and GEJ cancers [121–123]. Maintenance therapy using anti-PD-L1 (MEDI4736) in locally advanced and metastatic GEJ adenocarcinoma after the standard first-line treatment is currently being investigated in a phase II trial [124].

Ongoing clinical trials of checkpoint inhibitors are summarized in Table 1.

## 12. Conclusion

GC is a common malignancy with poor prognosis despite advances in surgical interventions and chemotherapy and radiotherapy techniques. Therefore, seeking novel treatment approaches is necessary. In this paper, we reviewed the recent studies on vaccination, on ACT, and on the use of checkpoint inhibitors in GC.

Vaccination is safe and tolerable and showed improvement in PFS and OS, especially when combined with chemotherapy. GC microenvironment is highly infiltrated with high cytolytic TILs with different recognition patterns towards GC antigens depending on their presentation in primary site, involved LNs, or metastatic sites. ACT in GC showed promising results in preclinical studies; it demonstrated tolerable side effects and antitumor cytotoxic efficacy against GC in both primary and metastatic sites. In clinical studies, ACT has a tolerable toxic profile, even in elderly patients, tumor reduction when administered either systemically or locally (intraperitoneal injection), and improved QoL and OS, especially when combined with conventional chemotherapy in both radically resected and advanced GC patients. However, more genetic and molecular studies are still needed to understand different pathognomic molecular expressions and distinguish which subtype of GC could be more sensitive to ACT. The PD-1/PD-L1 expression could be a prognostic factor in GC; however, results are controversial and it remains to be seen whether to consider high expression as a good prognostic factor or a poor one. Although clinical trials targeting PD-1/PD-L1 or CTLA-4 are, in most of cases, in phase I or II but with too few patients to make any conclusions, some updated results of ongoing clinical trials show promising results. Nevertheless, checkpoint inhibitor therapy provides a good safety profile in most cases, with modest antitumor response when combined with chemotherapy in advanced chemoresistant GC.

## Figures and Tables

**Table 1:** Ongoing clinical trials using the immune checkpoint inhibitors in GC.

Agent	Trial name/number	Phase	Trial population	Primary end points	Estimated study completion date
Nivolumab	A Study of Nivolumab by Itself or Nivolumab Combined With Ipilimumab in Patients With Advanced or Metastatic Solid Tumors/NCT01928394	I/II	Advanced solid tumors including GC	Overall response rate (ORR)	Dec-18

MEDI4736 Tremelimumab	A Phase 1b/2 Study of MEDI4736 With Tremelimumab, MEDI4736 or Tremelimumab Monotherapy in Gastric or GEJ Adenocarcinoma/NCT02340975	I-II	GC or GEJ Adenocarcinoma	ORR, PFS, and safety	17-Aug-18

Nivolumab/Ipilimumab	Efficacy Study of Nivolumab Plus Ipilimumab or Nivolumab Plus Chemotherapy Against Chemotherapy in Stomach Cancer or Stomach/Esophagus Junction Cancer (CheckMate649)/NCT02872116	III	GC or GEJ Adenocarcinoma	OS	11-Oct-20

ONO-4538 (Nivolumab)	Study of ONO-4538 in Unresectable Advanced or Recurrent Gastric Cancer/NCT02267343	III	Unresectable advanced or recurrent GC and GEJ adenocarcinoma	OS	Aug-17

MPDL3280A (Atezolizumab)	A Phase 1 Study of Atezolizumab (an Engineered Anti-Programmed Death-Ligand 1 [PDL1] Antibody) to Evaluate Safety, Tolerability and Pharmacokinetics in Participants With Locally Advanced or Metastatic Solid Tumors/NCT01375842	I	Locally advanced/metastatic solid tumor including GC	Dose limited toxicity	31-May-18

Nivolumab	An Investigational Immuno-therapy Study of Nivolumab or Placebo in Patients With Resected Esophageal or Gastroesophageal Junction Cancer (CheckMate 577)/NCT02743494	III	Resected esophageal and GEJ cancer	DFS/OS	1-Apr-21

Avelumab	Avelumab in Metastatic or Locally Advanced Solid Tumors (JAVELIN Solid Tumor)/NCT01772004	I	Metastatic or locally advanced solid tumors including GC and GEJ adenocarcinoma	Dose limiting toxicity/best overall response	31-May-18

Pembrolizumab (MK-3475)	A Study of Pembrolizumab (MK-3475) Versus Paclitaxel for Participants With Advanced Gastric/Gastroesophageal Junction Adenocarcinoma That Progressed After Therapy With Platinum and Fluoropyrimidine (MK-3475-061/KEYNOTE-061)/NCT02370498	III	Advanced GC and GEJ adenocarcinoma	PFS/OS	Aug-17

Pembrolizumab (MK-3475)	Study of Pembrolizumab (MK-3475) in Participants With Advanced Solid Tumors (MK-3475-012/KEYNOTE-012)/NCT01848834	I	Advanced solid tumors including GC	Adverse events	May-17

Avelumab	Avelumab in First-Line Maintenance Gastric Cancer (JAVELIN Gastric 100)/NCT02625610	III	Unresectable locally advanced/metastatic GC and GEC adenocarcinoma	OS	31-Mar-24

Avelumab	Avelumab in Third-Line Gastric Cancer (JAVELIN Gastric 300)/NCT02625623	III	Unresectable, recurrent, locally advanced, or metastatic GC and GEH adenocarcinoma	OS	30-Sep-22

ONO-4538 (Nivolumab)	Study of ONO-4538 in Gastric Cancer/NCT02746796	II	Unresectable advanced or recurrent GC and GEJ adenocarcinoma	ORR	Aug-20

Nivolumab/Ipilimumab	An Investigational Immuno-therapy Study to Investigate the Safety and Effectiveness of Nivolumab, and Nivolumab Combination Therapy in Virus-associated Tumors (CheckMate358)/NCT02488759	I/II	Virus-associated tumors including EBV GC	Drug related toxicity, ORR, and rate of surgery delay	Dec-19

Pembrolizumab	Pembrolizumab, Combination Chemotherapy, and Radiation Therapy Before Surgery in Treating Adult Patients With Locally Advanced Gastroesophageal Junction or Gastric Cardia Cancer That Can Be Removed by Surgery/NCT02730546	I/II	Unresectable locally advanced GC and GEJ adenocarcinoma	Pathological complete remission/PFS	Apr-18

Pembrolizumab	Pembrolizumab, Trastuzumab, HER2 Positive Gastric Cancer/NCT02901301	I/II	HER2 positive GC	ORR	Mar-18

Pembrolizumab (MK-3475)	Study of Pembrolizumab (MK-3475) as First-Line Monotherapy and Combination Therapy for Treatment of Advanced Gastric or Gastroesophageal Junction Adenocarcinoma (MK-3475-062/KEYNOTE-062)/NCT02494583	III	Advanced GC and GEJ adenocarcinoma	PFS/OS	6-Jun-20

Pembrolizumab (MK-3475)	Study of Pembrolizumab (MK-3475) Versus Investigator's Choice Standard Therapy for Participants With Advanced Esophageal/Esophagogastric Junction Carcinoma That Progressed After First-Line Therapy (MK-3475-181/KEYNOTE-181)/NCT02564263	III	EGJ adenocarcinoma	PFS/OS	31-Aug-18

Pembrolizumab (MK-3475)	A Study of Pembrolizumab (MK-3475) in Participants With Recurrent or Metastatic Gastric or Gastroesophageal Junction Adenocarcinoma (MK-3475-059/KEYNOTE-059)/NCT02335411	II	Advanced GC and GEJ adenocarcinoma	Drug related toxicity/ORR	Jun-18

MEDI4736	Planning Treatment for Oesophago-gastric Cancer: a Maintenance Therapy Trial (PLATFORM)/NCT02678182	II	Locally advanced or metastatic HER2 positive or HER2 negative oesophagogastric adenocarcinomas	PFS	Feb-20
